# Improving Nutrition Knowledge and Skills by the Innovative Education Program MaestraNatura in Middle School Students of Italy

**DOI:** 10.3390/nu14102037

**Published:** 2022-05-12

**Authors:** Rosaria Varì, Antonio d’Amore, Annalisa Silenzi, Flavia Chiarotti, Sara Del Papa, Claudio Giovannini, Beatrice Scazzocchio, Roberta Masella

**Affiliations:** 1 Center for Gender-Specific Medicine, Italian National Institute for Health, 00161 Rome, Italy; rosaria.vari@iss.it (R.V.); antonio.damore@iss.it (A.d.); annalisa.silenzi@gmail.com (A.S.); sara.delpapa8@gmail.com (S.D.P.); claudio.giovannini@iss.it (C.G.); roberta.masella@iss.it (R.M.); 2 Reference Center for Behavioral Sciences and Mental Health, Italian National Institute for Health, 00161 Rome, Italy; flavia.chiarotti@iss.it

**Keywords:** knowledge, skills, education, nutrition, school, students, healthy lifestyle

## Abstract

Promoting a healthy diet, mainly in youths, is the most effective action to prevent and fight dietary excesses and nutritional imbalance in the population. MaestraNatura (MN) is an innovative nutritional education program aimed at promoting a healthy lifestyle in first-level secondary school students. The study evaluated the effectiveness of the MN program in improving knowledge in students following the MN program (MN group) with respect to a control group (CO group) undergoing a “traditional” nutritional education path. To this end, the nutrition knowledge of the two groups was assessed by three multi-choice questionnaires. The results showed a significant improvement in knowledge (*p* < 0.001) in the MN group with respect to the CO group for all the questionnaires. Furthermore, the students’ ability to transfer the principles of nutrition guidelines to the real context of daily meals was determined by asking the MN group to create a weekly food plan before (T0) and after (T1) the completion of the MN program. The MN group demonstrated improved performance in organizing the weekly menu plan at T1 with respect to T0 (*p* = 0.005). In conclusion, the MN nutritional education program appears to be an effective tool for improving knowledge and skills on nutritional issues, especially in those students with a lower starting level of knowledge and ability.

## 1. Introduction

During the past few decades, many countries of the WHO European Region have reported rising rates of overweight and obesity, which are persistently high and accelerating particularly among children and adolescents of southern countries [1]. Recent data from Italy show that more than 30 percent of children and adolescents are overweight or obese, highlighting a clear geographical trend across the southern regions, showing a higher percentage of overweight/obesity with respect to the northern ones [2]. This is happening despite the abundance and widespread dissemination of guidelines for healthy nutrition both in the US and Europe [3,4], which is evidence of the scarce influence that mere information can exert on the modification of behavioral patterns. In 2008, the European Parliament resolution on the “White Paper on nutrition-, overweight-, and obesity-related health issues” indicated a multilevel and comprehensive approach for the best way to fight obesity among the EU population. The resolution highlighted the importance of actions to improve the Health Literacy of citizens and the need for a broader educational strategy, for example, by means of lessons on diet and health in primary schools [5]. Increasing knowledge and interest about food and nutrition are typical strategies for nutrition intervention and are important prerequisites for eliciting diet-related behavioral changes [6]. Indeed, the school represents the ideal setting for interventions that support healthy behaviors [7], which can potentially reach most children of diverse ethnic and socioeconomic groups [8]. Moreover, several studies have indicated the family as the basic unit for promoting healthy habits [9], whilst also indicating interventions that integrate behavioral and social-learning theories, as well as school and family, to be effective tools for eliciting healthy behaviors in children. However, school-based nutritional education interventions usually fail to target and involve the families [10,11]. Of note, introducing the internet into clinical practice as a tool for sharing information has brought many opportunities for innovative interventions [12]. A recent meta-analysis of the effectiveness of web-based versus non-web-based interventions revealed significant improvements in knowledge acquisition or behavior outcome when web-based programs were used [13]. Based on these premises, educational interventions relying on behavioral theory; the involvement of school and family to support the activities of children, teachers, and parents; and the use of the internet should be considered as the best strategies for increasing the effectiveness of obesity prevention interventions. In Italy, a new nutritional education program that supports these implications, the MaestraNatura Program (MNP), was developed to guide children through an 8-year learning path whilst attending primary and first-level secondary schools [14]. MNP is inspired by both social learning theory [15] and experiential learning theory [16]. All topics covered by MNP, concerning food, nutrition, healthy eating, and a sustainable diet, are integrated into the school curricula in order to facilitate teachers in their work. The MNP takes advantage of an internet platform that supports teachers and parents in organizing all the activities aiming to eventually encourage the family to adopt healthier dietary habits. Indeed, improvement in knowledge and skills is a necessary step to favor behavioral changes and the acquisition of healthy eating behaviors [6]. The aim of the study was to compare the effectiveness of the MNP path “We Are What We Eat” in improving knowledge about food and nutrition in children with respect to the usual nutritional education intervention. The study also aimed to examine whether the nutritional education intervention was able to favor the acquired knowledge and skills being transferred into a real context, such as planning a healthy weekly menu.

## 2. Materials and Methods

### 2.1. Participants

**Figure 1:**
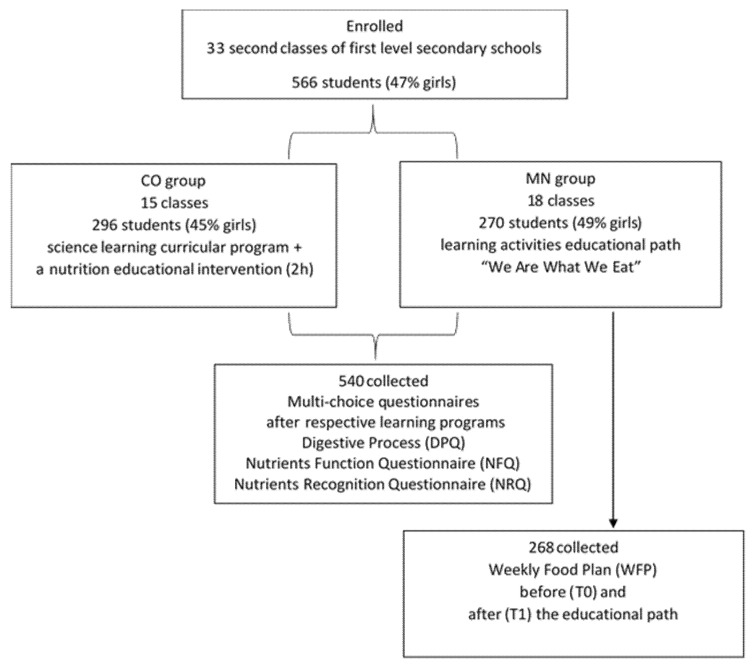
Flow chart of the study and subject recruitment.

Second-year classes of first-level secondary public schools were enrolled in the study. The schools were located in three distinct geographic and socioeconomic Italian areas: Veneto (small towns, to the north), Lazio (small and big towns, in the center), and Basilicata (small towns, to the south). A total number of 566 students (294 boys, 272 girls), aged 11–13 years, participated in the study. The classes were randomly allocated either to the control group (CO) (161 boys, 135 girls) or to the MaestraNatura group (MN) (133 boys, 137 girls) (Figure 1).

### 2.2. Ethical Aspects

The data were collected according to the parental written informed consent obtained for the participation of their children, in agreement with both ethical and legal (personal data protection) requirements of Italian law. The study was explained to the participants before the start of the education activities, by meeting with the teachers and providing leaflets to the parents through the schools. The study was approved by the ethics committee of Istituto Superiore di Sanità (AOO-ISS 26.04.21 n.0015951).

The data collected were pseudonymised soon after data quality control. A univocal numerical code was assigned to each subject in order to allow the connection of data collected on the same subject before and after the execution of the educational program.

### 2.3. Procedure

The CO group underwent a ‘traditional’ nutritional education program, attending curricular science lessons and one lesson (2 h) focused on the basic nutrition principles of a balanced diet as indicated by the Food Pyramid, held by an expert nutritionist [17]. The MN group participated in all the learning activities provided by the MNP educational path “We Are What We Eat”. The path included three didactic power point presentations (“The digestive process”, “There is no perfect food”, and “Recognizing nutrients”) and experimental and practical activities aimed at increasing knowledge on nutrients, food, the digestive process, and the importance of a healthy and varied diet (“Simulating the digestion process”, “Discover macronutrients providing energy”, “What’s inside?”, and “How many times a day, how many times a week?” [14]). In addition, many recipes on how to cook vegetables, fruit, cereals, and legumes were provided to be carried out at home in order to favor interactions between children and their parents and to encourage children to experience new tastes. All the planned activities were carried out throughout the school year. All the didactic contents were downloadable from the MN web platform, divided into three areas specifically dedicated to teachers, parents, and students.

In order to test knowledge levels, at the end of their respective learning programs, CO and MN groups were required to fill in three multiple-choice questionnaires: the Digestive Process Questionnaire (DPQ), the Nutrients Function Questionnaire (NFQ), and the Nutrients Recognition Questionnaire (NRQ). The questionnaire contents are shown in Table 1.

Furthermore, the MN group was tested to assess their ability in transferring the knowledge acquired by the nutritional education path to the context of daily life. The students were asked to organize a Weekly Food Plan (WFP) both before (T0) and after (T1) completion of the educational activities. The WFP requires the construction of a complete daily menu, composed of breakfast, snack-1, lunch, snack-2, and dinner, for each day of the week. The menu was constructed by each child gluing stamps bearing the figures of the food belonging to the different food groups. Each menu was evaluated on the basis of the following criteria: daily insertion of breakfast, servings of fruit and vegetables, weekly servings of legumes/cereals and fish, and correct use of protein food, in order to maintain the adequate quantities and frequencies of food in the meals. A total score was calculated by counting the number of breakfasts, servings of fruit, vegetables, fish, cereals, and legumes included in the weekly menu, and by penalizing the excessive insertion of protein-rich food. Individual scores were calculated by taking into account, separately, the number of servings of fruit and vegetables, respectively. This allowed us to compare the scores totalized at T0 and T1 by each student and to assess the improvement of children’s performance, if there was any. On the basis of the scores, the students were assigned to one of three categories of a rating scale indicative of different levels of performance (low, <20; medium, 21–27; and high >28 points).

### 2.4. Statistical Analysis

Quantitative variables were summarized using means, standard deviation, medians, and ranges. Categorical variables were synthesized by absolute and percent frequencies. Regarding the three questionnaires, for any item, the answer given by a single child was categorized as correct (1 out of 4 possible answers) or incorrect (3 out of 4 possible answers), and for both groups, the actual proportion of children was tested against the theoretical expected proportion of correct answers corresponding to the chance value (chance proportion = 0.25). Considering the proportion of children giving the correct answers, for any single item, the difference between CO and MN groups was assessed by the Fisher’s exact probability test. Moreover, the difference between the proportions of subjects giving the correct answer in MN and in CO groups (propMN-propCO) was computed, and Cohen’s h index was used to measure the effect size of the difference between these proportions (h = absolute(arcsin(sqrt(propMN))-arcsin(sqrt(propCO))). Therefore, a difference between CO and MN groups was considered as reasonably significant only when (i) the Fisher’s exact probability test showed a statistically significant difference (*p* < 0.05), and (ii) Cohen’s h index was higher than 0.20. Moreover, the global correctness index (GCI) of answers to the questionnaire was calculated as the proportion of items receiving the correct answer on the whole questionnaire. Differences between the two learning program groups with respect to the GCI were assessed by the Mann–Whitney U test. Regarding the evaluation of WFP activity, variation between scores at T0 and T1 was analyzed by Student’s t test. The ANOVA test, followed by post-hoc tests, was used when > 2 groups were compared. Differences were considered significant when *p* values were < 0.05. The Chi-squared test was used in intergroup comparisons of non-parametric variables, and data were expressed as percentages. All statistical analyses were performed by STATA 16.0.

## 3. Results

### 3.1. Evaluation of the Improvement in Knowledge Obtained by “We Are What We Eat” Path with Respect to a ‘Traditional’ Nutritional Education Intervention

The questionnaires filled in by 540 students (corresponding to about the 95% of the total students enrolled) were collected and analyzed. The MN group showed a proportion of correct answers greater than the CO group for all items of all the three questionnaires. Differences were statistically significant for most of the items, except for item 2 of DPQ, the items 1 and 9 of NFQ, and items 8 and 17 of NRQ (Table 2a–c).

On average, the proportion of correct answers for the questionnaire was significantly higher in the MN compared to the CO group for all the questionnaires (DPQ: mean = 0.60, SD = 0.22 vs. mean = 0.44, SD = 0.19, *p* < 0.001; NFQ: mean = 0.67, SD = 0.20 vs. mean = 0.49, SD = 0.21, *p* < 0.001; NRQ: mean = 0.72, SD = 0.18 vs. mean = 0.57, SD = 0.16; *p* < 0.001) (Table 3). The observed effects were independent of gender, region, and town size for all questionnaires (data not shown).

### 3.2. Skill Improvement in Planning a Weekly Menu

In total, 268 students completed a WFP before (T0) and after (T1) completing the “We Are What We Eat” path. Nearly 60% of the students showed an improvement in planning the WFP. The students that enhanced their performance were distributed on the basis of scores achieved both at T0 and T1 into three categories of the rating scale considered indicative of different levels of performance, as shown in Table 4. It is extremely interesting to see that the percentage of students in the lowest category was 37% at T0, but only about 7% at T1. In addition, a significant increase in the percentage of students in the category with the highest score (about 29% and 57% at T0 and T1, respectively) was shown. Furthermore, the percentage of students in the category of the highest score, assessed in every single region and in Rome and the province of Rome separately, resulted in significant increases at T1 in all of them (Table 4).

Regarding the comparison between the total score obtained planning the weekly menu, a significant increase in the total score was observed at T1 with respect to T0 (*p* = 0.005) (Table 5). Moreover, separately taking into account the score obtained for the servings of fruit and vegetables included in the food plan, a significant increase in score was observed (*p* = 0.042 and *p* = 0.006 for fruit and vegetables, respectively) (Table 5). In contrast, no significant differences between T0 and T1 were determined in the scores obtained for the inclusion of breakfast and servings of fish, cereals, and legumes (data not shown). Taken individually with respect to the distinct geographic areas and the metropolitan city of Rome, the data collected demonstrated that the students from Rome demonstrated better performance with respect to the others (Table 5) at T0, while those living in small towns of the province of Rome and Basilicata demonstrated worse performance; however, these last students achieved the greatest improvement at T1 (*p* = 0.016, *p* < 0.001, respectively), which was essentially due to the increase in scores obtained for the number of servings of fruit (*p* = 0.026; *p* = 0.001 for the province of Rome and Basilicata, respectively) and vegetables (*p* = 0.018, the province of Rome) (Table 5).

Finally, by analyzing the results by sex, some differences were highlighted between boys and girls (Table 6). Males showed a worse score at T0 with respect to females (*p* = 0.005), especially due to the small number of fruit (*p* = 0.011) and vegetable (*p* = 0.010) servings included during the week; although the boys improved their ability at T1, the difference from girls remains statistically significant (*p* = 0.021) (Table 6).

## 4. Discussion

This study carried out in students (aged 11–13 years old) attending the second year of first-level secondary schools demonstrated the effectiveness of the educational path “We Are What We Eat” in increasing knowledge on food and nutrition, and in improving children’s ability to plan a complete weekly menu following the nutritional guidelines for a healthy diet. The nutritional knowledge of adolescents is often inadequate, and findings suggest that its increase is an important prerequisite for achieving changes in dietary behavior. A systematic review revealed that most of the 29 studies analyzed reported a positive association between nutritional knowledge and dietary intake quality, especially regarding the intake of vegetables and fruit [18]. Another systematic review highlighted the association between food literacy and adolescents’ dietary habits, strongly supporting the idea that food literacy may have a role in defining eating behaviors [19].

The term ‘food literacy’ brings together nutritional knowledge and skills and the ability to make critical decisions about dietary intake according to nutritional guidelines [20]. Indeed, interventions only focused on improving nutritional knowledge seem to be not very effective in promoting positive healthy habits [21]. In contrast, promoting food literacy in the population could be the right tool to gain effective results [22].

The present study showed that MN may represent a useful program for improving both nutritional knowledge and skills related to food choices and processing. In this study, we observed a significant improvement in knowledge on the digestive process and nutrient functions and recognition in students following MNP with respect to those attending science lessons and receiving a traditional nutritional education intervention. It must be emphasized that better results were systematically obtained by the MN group in all the items as evidenced by the increase in the degree of correctness of the answers to the three questionnaires with respect to the CO group. The few questions that failed to demonstrate better performance of the MN group were those presenting a high percentage of correct answers in both the groups, most likely because they were much too easy questions/answers. Indeed, when the questions concerned more complex topics, the greatest difference in the percentages of correct answers between the groups was observed. This indicated that the “We Are What We Eat” path of MNP was effective, specific, and also consistent with previous nutritional education interventions implemented in the school setting among adolescents [6]. Further supporting these results, the ‘chance’ effect was evidenced only for a few questions and mainly in the CO group; in the MN group, only one question showed this effect, probably because it dealt with a difficult and very specific issue such as the place where the digestive process of simple sugars occurs.

Regarding the WFP task, by comparing the total scores obtained at T0 and T1 by the students that followed the MNP path, a significant increase (*p* = 0.005) in the ability to plan a weekly menu was evidenced. The improvement was particularly evident for the students living in small towns; they were able to overcome the initial gap with the students from Rome, reaching an appreciable level of knowledge at the end of the didactic path. A similar result was obtained when considering the different performances between girls and boys. The boys achieved worse scores with respect to girls, but after the educational program, their performances improved significantly. These results do not conflict with the effectiveness of the program, but rather confirm that the MN program is especially effective in those students with a lower starting level of knowledge and ability. Although the menu plan did not represent a food diary, most likely, at T0, children could have been influenced by their own eating habits. Thus, the lower performance observed at T0 among children from Basilicata, Veneto, and the province of Rome could in part reflect the unhealthy eating habits common in provincial areas. A study evaluating the eating habits of a large sample of children living in 19 European countries showed that in both urban and rural areas, eating behaviors were not optimal and required improvement. However, in some countries in Eastern and Southern Europe, including Italy, children attending schools located in rural areas were more likely not to eat fruit and vegetables daily compared to children living in urban areas [23]. Furthermore, our findings evidence that children living in Rome have better basic knowledge about the correct intake of food than those living in small towns (T0, *p* < 0.001 vs. Basilicata and the province of Rome, *p* = 0.006 vs. Veneto). This allowed them to demonstrate better performance in planning a weekly menu with respect to the other children, suggesting a possible influence of the socio-cultural environment.

Thus, the “We Are What We Eat” path might improve nutritional knowledge mainly in children living in provincial environments, significantly increasing their ability to select healthier foods, supporting the hypothesis that good levels of nutritional knowledge are associated with the ability to make healthy food choices. Altogether, the data seem to confirm that the MNP experiential learning approach is effective in improving nutritional knowledge and skills among school children, in agreement with previous results obtained by other intervention studies. In particular, a systematic review demonstrated that enhanced curricula, cross-curricula, and experiential learning approaches are the most effective teaching strategies in leading to positive changes in primary school children’s nutritional knowledge and behaviors [24]. In this regard, it is worth noting that the studies recording significant improvements in fruit preference or consumption have taken advantage of curriculum-based approaches coupled with secondary ones (e.g., experiential learning, parental involvement) [25]. One of the most relevant aspects of the didactical path “We Are What We Eat” was the concomitant involvement of teachers and parents. Many practical activities were implemented to be carried out both at school (experiments) and at home (cooking). The realization of recipes at home was considered as an extension of didactic activity, not only able to strengthen what was learned at school but also able to remove the students’ unwillingness to consume certain foods such as fruit, vegetables, and legumes. Indeed, a child is more motivated to experiment with new food and more interested in tasting it as the result of something that he/she has helped to create together with parents. This is a relevant aspect of the study since a recent literature review demonstrated that adolescents living in North America, Europe, or Oceania are far from being compliant with the nutritional recommendations for fruit, vegetables, legumes, and sodium, and they do not follow the principles of the Mediterranean Diet [26].

Taken together, this study indicates that the path “We Are What We Eat” of MNP, independently of gender, region, and size of town, significantly improves nutritional knowledge and the ability to select healthier foods in a weekly planning task.

## 5. Conclusions

Considering the high digital divide currently present in our country [27], the MaestraNatura internet platform seems to be a suitable means for guaranteeing participation continuity for all three actors (e.g., teachers, students and parents) involved in the program. However, future developments of the program should consider greater involvement of the family, including more activities to be done at home and evaluating their efficacy in favoring healthier eating habits of the family.

Furthermore, the path “We Are What We Eat” of MNP effectively addresses all the characteristics suggested by the EU ‘School fruit and vegetable scheme’, which aims to favor the consumption of fruit and vegetables by children, whilst also taking advantage of educational measures, including lessons, farm visits, school gardens, tasting, and cooking [28,29].

Moreover, although the MNP initially addressed Italian students, it could be adapted to other geographical and socio-cultural contexts. In fact, all the contents can be easily translated to any language, and the included nutritional principles are those reported by almost all the nutritional guidelines inspired by the Mediterranean diet. In conclusion, the MNP program may serve as a first step for the implementation of educational programs and preventive strategies for public health, able to favor the adoption of healthier eating behaviors by young people.

## Figures and Tables

**Table 1:** Multi-choice questionnaires used to assess students’ knowledge on different nutrition-related issues.

**Digestive Process Questionnaire (DPQ)**
1. The digestion of complex carbohydrates (or starch) begins…
2. The digestion of simple sugars takes place…
3. What is the function of secretion in the digestive process?
4. What does absorption mean?
5. Which of the following glands / organs is NOT related to digestion?
6. Which of the following functions is NOT performed by the stomach?
7. Which of the following functions is NOT performed by the small intestine?
8. Which of the following functions is NOT performed by the large intestine?
9. Which organ produces bile?
10. Where is bile stored?
11. Which of these functions is NOT performed by the liver?
12. Which of these functions is NOT performed by the pancreas?
**Nutrient Function Questionnaire (NFQ)**
1. What function does water perform in the body?
2. What is the main function of carbohydrates?
3. Which of the following nutrients is used less by the body to produce energy?
4. Which of these functions is NOT performed by fats?
5. Which of the following nutrients does NOT provide energy?
6. What function do vitamins have?
7. Which of the following carbohydrates provides energy more quickly?
8. Which of the following carbohydrates provides the lowest amount of energy?
9. Which of the following nutrients, at the same weight, provides energy more quickly ?
10. Which of the following nutrients, at the same weight, provides more energy?
11. What are essential amino acids?
**Nutrient Recognition Questionnaire (NRQ)**
1. Which of the following foods, at the same weight (e.g., 100 g), contains more water?
2. Which of the following foods, at the same weight (e.g., 100 g), contains less water?
3. Which of the following foods, at the same weight (e.g., 100 g), provides more energy?
4. Which of the following foods, at the same weight (e.g., 100 g), provides more energy?
5. Which of the following foods, at the same weight (e.g., 100 g), contains less protein?
6. Which of the following foods does NOT contain carbohydrates?
7. Do cereals (wheat, barley, spelled, rye, etc.) contain protein?
8. Which of the following foods does NOT contain fats?
9. Which of the following foods, at the same weight (e.g., 100 g), is higher in fat?
11. Which of the following foods, at the same weight (e.g., 100 g), contains less saturated fat?
12. Which of the following foods is NOT a cereal?
13. Which of the following foods is NOT a legume?
14. A portion of rice and peas can replace…
15. A portion of pasta and beans can replace…
16. A portion of potatoes can replace…
17. Can a portion of milk be replaced by a portion of yoghurt?
18. Fats can also be called…
19. Carbohydrates can also be called…
20. Fats can be…
21. Which of these substances is NOT a food?
22. Which of these substances is NOT a nutrient?

**Table 2:** Proportion of students giving the correct answers to the various questions of the three questionnaires.

(**a**) Digestive process questionnaire (DPQ)							
**Item**	**Proportions**			**Binomial *p***		**Fisher *p***	**Cohen’s h**
	**CO**	**MN**	**MN-CO**	**CO**	**MN**	**MN vs. CO**	
1	0.4545	0.6085	0.1540	<0.001	<0.001	<0.001	0.31
2	0.2182	0.2597	0.0415	0.44	0.19	0.27	0.10
3	0.4473	0.7016	0.2543	<0.001	<0.001	<0.001	0.52
4	0.6291	0.7984	0.1694	<0.001	<0.001	<0.001	0.38
5	0.7345	0.8837	0.1492	<0.001	<0.001	<0.001	0.39
6	0.2655	0.3463	0.0808	0.15	<0.001	0.05	0.18
7	0.3127	0.4784	0.1657	0.005	<0.001	<0.001	0.34
8	0.3200	0.4961	0.1761	0.002	<0.001	<0.001	0.36
9	0.5964	0.6899	0.0936	<0.001	<0.001	0.03	0.20
10	0.5709	0.7549	0.1840	<0.001	<0.001	<0.001	0.39
11	0.4036	0.5504	0.1468	<0.001	<0.001	0.001	0.29
12	0.3504	0.6008	0.2504	<0.001	<0.001	<0.001	0.51
(**b**) Nutrient function questionnaire (NFQ)							
**Item**	**Proportions**			**Binomial *p***		**Fisher *p***	**Cohen’s h**
	**CO**	**MN**	**MN-CO**	**CO**	**MN**	**MN vs. CO**	
1	0.7169	0.7647	0.0478	<0.001	<0.001	0.24	0.11
2	0.7426	0.8672	0.1245	<0.001	<0.001	<0.001	0.32
3	0.3824	0.6568	0.2745	<0.001	<0.001	<0.001	0.56
4	0.2831	0.5515	0.2684	0.06	<0.001	<0.001	0.55
5	0.5515	0.7721	0.2206	<0.001	<0.001	<0.001	0.47
6	0.4081	0.6852	0.2771	<0.001	<0.001	<0.001	0.56
7	0.6581	0.7897	0.1316	<0.001	<0.001	0.001	0.30
8	0.5294	0.6974	0.1680	<0.001	<0.001	<0.001	0.35
9	0.5478	0.5535	0.0057	<0.001	<0.001	0.93	0.01
10	0.2610	0.4170	0.1559	0.18	<0.001	<0.001	0.33
11	0.3493	0.6015	0.2522	<0.001	<0.001	<0.001	0.51
(**c**) Nutrient recognition questionnaire (NRQ)							
**Item**		**Proportions**		**Binomial *p***		**Fisher *p***	**Cohen’s h**
	**CO**	**MN**	**MN-CO**	**CO**	**MN**	**MN vs. CO**	
1	0.5625	0.7935	0.2310	<0.001	<0.001	<0.001	0.50
2	0.3971	0.6883	0.2912	<0.001	<0.001	<0.001	0.59
3	0.1618	0.3320	0.1702	0.50	0.001	<0.001	0.40
4	0.2390	0.3563	0.1173	0.32	<0.001	0.004	0.26
5	0.4412	0.6721	0.2309	<0.001	<0.001	<0.001	0.47
6	0.2684	0.4980	0.2296	0.13	<0.001	<0.001	0.48
7	0.5662	0.6842	0.1180	<0.001	<0.001	0.007	0.24
8	0.8419	0.8988	0.0569	<0.001	<0.001	0.07	0.17
9	0.6949	0.7814	0.0865	<0.001	<0.001	0.03	0.20
10	0.5441	0.6761	0.1320	<0.001	<0.001	0.002	0.27
11	0.5846	0.7045	0.1199	<0.001	<0.001	0.005	0.25
12	0.7574	0.8462	0.0888	<0.001	<0.001	0.01	0.22
13	0.8676	0.9393	0.0716	<0.001	<0.001	0.007	0.25
14	0.3529	0.4656	0.1126	<0.001	<0.001	0.01	0.23
15	0.5919	0.8138	0.2219	<0.001	<0.001	<0.001	0.49
16	0.5772	0.7126	0.1353	<0.001	<0.001	0.001	0.28
17	0.7353	0.7895	0.0542	<0.001	<0.001	0.15	0.13
18	0.7059	0.8502	0.1443	<0.001	<0.001	<0.001	0.35
19	0.5588	0.7449	0.1861	<0.001	<0.001	<0.001	0.39
20	0.7279	0.8623	0.1344	<0.001	<0.001	<0.001	0.34
21	0.8125	0.9150	0.1025	<0.001	<0.001	0.001	0.30
22	0.5772	0.7247	0.1475	<0.001	<0.001	<0.001	0.31

Differences between the control group (CO) and MaestraNatura (MN) group that participated in all the learning activities provided by the MNP educational path “We Are What We Eat” (MN) were considered significant when (i) the Fisher’s exact probability test showed a statistically significant difference (*p* < 0.05) and (ii) Cohen’s h index was >0.20.

**Table 3:** Degree of correctness in compiling the different questionnaires. The table shows a global correctness index (proportion from 0 to 1) of answers to the Digestive process (DPQ), Nutrient function (NFQ), and Nutrient recognition (NRQ) questionnaires calculated as the ratio between the number of questions receiving the correct answers and total number of questions of the questionnaire.

Questionnaire	CO Group		MN Group		Mann–Whitney U *p*
	*n*	Mean (SD)	*n*	Mean (SD)	
DPQ	273	0.44 (0.19)	254	0.60 (0.22)	<0.001
NFQ	271	0.49 (0.21)	269	0.67 (0.20)	<0.001
NRQ	271	0.57 (0.16)	246	0.71 (0.18)	<0.001

Comparison between control (CO) and MaestraNatura (MN) groups by Mann–Whitney U test; *p* < 0.05 was considered significant. SD—standard deviation.

**Table 4:** Classification of the students that improved their performance in planning the weekly menu in three categories of a rating scale. The students that improved their performance at T1 with respect to T0 were assigned to one of three categories of a rating scale considered indicative of different levels of performance (low, ≤20; medium, 21–27; and high, ≥28 points) on the basis of the scores obtained at T0 and T1.

	All			Basilicata			Province of Rome			Rome			Veneto		
SCORE	T0	T1	*p*	T0	T1	*p*	T0	T1	*p*	T0	T1	*p*	T0	T1	*p*
≤20	36.9	7.4	0.011	9.4	2.0	0.022	14.1	3.4	0.041	7.4	0.7	0.002	6.0	1.3	0.020
21–27	33.6	36.2	n.s.	8.7	9.4	n.s.	4.7	8.7	n.s.	14.1	13.4	0.014	6.0	4.7	n.s
≥28	29.5	56.4	<0.001	2.7	9.4	0.031	8.1	14.8	0.050	11.4	18.8	0.025	7.4	13.4	0.020

Data are presented as percentages of the students assigned to each category. n.s. not significant. Chi-squared test was used in intergroup comparisons. *p* < 0.05 was considered significant.

**Table 5:** Weekly Food Plan (WFP) evaluation. Total score and single score considering vegetable and fruit servings independently, obtained by the students by compiling a weekly menu plan before (T0) and after (T1) completing the educational path “We Are What We Eat” of the MaestraNatura Program (MNP).

	T0 Mean (SD)	T1 Mean (SD)	*T1-T0*	*p*
Total				
All	25.72 (8.24)	27.10 (7.5)	1.38	0.005
Basilicata	21.87 (6.87)	25.4 (6.42)	3.52	0.001
Veneto	25.75 (6.85)	27.83 (6.8)	2.07	0.027
Province of Rome	22.72 (8.72)	25.47 (9.5)	2.76	0.016
Rome	29.33 (7.60)	28.56 (7.48)	−0.83	0.450
Vegetables				
All	7.52 (4.15)	8.15 (3.74)	0.63	0.006
Basilicata	5.27 (2.98)	5.4 (3.15)	0.13	0.783
Veneto	7.61 (3.93)	8.79 (3.29)	1.18	0.018
Province of Rome	7.19 (4.55)	8.53 (4.1)	1.34	0.018
Rome	8.64 (3.98)	8.68 (3.52)	0.04	0.687
Fruit				
All	9.74 (4.93)	10.4 (4.98)	0.066	0.042
Basilicata	8.22 (4.28)	11.07 (4.09)	2.85	0.001
Veneto	9.75 (4.47)	10.51 (4.43)	0.76	0.236
Province of Rome	7.50 (4.67)	9.01 (5.6)	1.51	0.026
Rome	11.80 (4.71)	11.06 (5.00)	−0.74	0.209

The table reports the means ± SD of the scores obtained by the students by creating a weekly menu plan. The *total* score was calculated by counting the number of breakfasts, servings of fruit, vegetables, fish, cereals, and legumes and penalizing wrong inclusion of protein-rich food. *Vegetables* and *Fruit* represent the scores reported taking into account only the total servings of vegetables and fruit added to the menu, respectively. Variation between the score at T1 and T0 was calculated by Student’s t Test. *p* < 0.05 was considered significant.

**Table 6:** Total and individual scores for vegetables and fruit disaggregated by sex.

Total			
	T0 Mean (SD)	T1 Mean (SD)	*p* (T0 vs. T1)
F	27.08 (8.11)	28.19 (7.54)	n.s.
M	24.23 (8.13)	25.99 (7.59)	n.s.
*p* (F vs. M)	0.005	0.021	
Vegetables			
F	8.13 (3.88)	8.66 (3.32)	n.s.
M	6.84 (4.34)	7.61 (4.1)	0.076
*p* (F vs. M)	0.011	0.022	
Fruit			
F	10.45 (4.74)	10.9 (5.11)	ns
M	8.96 (5.03)	9.94 (4.75)	0.023
*p* (F vs. M)	0.011	ns	

The table reports the means ± SD of the scores obtained by the students by creating a weekly menu plan. The total score was calculated by counting the number of breakfasts, servings of fruit, vegetables, fish, cereals, and legumes and penalizing wrong inclusion of protein-rich food. Vegetables and Fruit represent the scores reported taking into account only the total servings of vegetables and fruit added to the menu, respectively. Variation between the scores at T1 and T0 was calculated by Student’s t Test. n.s., not significant. *p* < 0.05 was considered significant.

## Data Availability

The data presented in this study are available on request from the corresponding author.
